# Urban–rural background disparities and their impact on college students' academic outcomes

**DOI:** 10.3389/fpsyg.2026.1724706

**Published:** 2026-02-16

**Authors:** Linchuan Wang, Longlong Pang, Sijia Yang, Haiyue Shui

**Affiliations:** 1 School of Economics, Hefei University, Hefei, China; 2 School of Management, Hefei University, Hefei, China

**Keywords:** academic outcomes, college students, educational equity, social reproduction, urban–rural disparities

## Abstract

**Background:**

In recent years, increasing attention has been devoted to the influence of urban–rural background disparities on students' educational outcomes. Focusing on undergraduate students with comparable performance on the National College Entrance Examination, this study examines whether urban–rural background disparities remain associated with academic outcomes in higher education in China.

**Methods:**

Using data from 632 valid questionnaire responses, we construct a mediation model incorporating family economic conditions, parental educational attainment, and self-directed learning ability to explore potential associational pathways linking urban–rural background to academic outcomes.

**Results:**

The results show that, on average, students from urban backgrounds achieve significantly better academic outcomes than their rural counterparts. Further analyses indicate that urban–rural background is systematically associated with differences in family economic conditions, parental educational attainment, and self-directed learning ability, which are in turn statistically associated with academic outcomes and together account for a substantial portion of the observed disparities.

**Conclusions:**

By embedding urban–rural background disparities into an analytical framework of college students' academic outcomes and jointly considering family-, individual-, and societal-level factors, this study extends the existing literature. Within the limits of a cross-sectional observational design, the findings clarify the associational structure linking family background, individual learning characteristics, and academic performance at the university level, and offer policy-relevant insights for improving learning conditions for rural students and promoting educational equity.

## 1 Introduction

Educational equity constitutes both an independent objective in the United Nations' Sustainable Development Goals and a foundational condition for poverty reduction, equality promotion, and sustainable socio-economic development (Ainscow, 2020). Since the establishment of China's household registration (hukou) system in the 1950s', the urban–rural dual structure has become increasingly entrenched, shaping population mobility, access to welfare, and the distribution of educational opportunities (Wu, 2011). The hukou system has long restricted rural residents' access to urban public services (Ruan, 2024), thereby reinforcing significant disparities in economic development, resource allocation, and educational opportunities between urban and rural regions (Xu and Wu, 2022; Ruan, 2024). Against this institutional backdrop, questions regarding educational equity have become particularly salient.

Despite rapid economic growth over the past four decades, access to educational resources in China remains highly uneven across regions and demographic groups (Zou and Ma, 2019). Educational equity is not only a pillar of social fairness but also a key component of China's pursuit of common prosperity and modernization. The 20th National Congress of the Communist Party of China explicitly calls for expanding educational equity, promoting the sharing of high-quality educational resources, and narrowing urban–rural, regional, and group-based gaps. This policy stance highlights the strategic importance of education and underscores the enduring challenges associated with urban–rural disparities. Concurrently, public discourse on educational mobility has shifted markedly—from the once-celebrated notion of “rural students rising through hard work” to the more pessimistic view that “children from poor families face limited opportunities” (Ping and Huang, 2024; Bian, 2025). This discursive shift reflects persistent inequalities in family resources, educational conditions, and developmental opportunities. Yet the mechanisms through which these disparities translate into unequal learning outcomes remain insufficiently understood, calling for systematic inquiry into their pathways of influence.

Research in developed countries presents a more complex and sometimes contradictory picture. Some studies report minimal academic achievement gaps between urban and rural students across OECD systems; in contexts such as the United States and the United Kingdom, rural students may even outperform their urban peers, possibly due to smaller class sizes, close-knit communities, and relatively stable family conditions (Echazarra and Radinger, 2019; Cherry, 2021). Nevertheless, other evidence points to persistent structural challenges in rural schooling, including limited technological resources (Farrington et al., 2015), difficulties recruiting and retaining high-quality teachers (Ovenden-Hope and Passy, 2019), and geographic isolation that constrains inter-school collaboration (Muijs, 2015). Scholars also emphasize that rural students in developed contexts may experience long-term disadvantages not immediately visible in cross-sectional comparisons (Midouhas and Flouri, 2015; Davies et al., 2021), including sustainability issues in resource provision and teacher workforce development (Cao and Huo, 2025).

In developing countries, urban–rural disparities are more pronounced and persistent. Evidence from Peru and India shows that rural students face substantial disadvantages throughout basic education (Agrawal, 2014; Castro and Rolleston, 2015; Gorard et al., 2022). Analyses using Young Lives data indicate that cognitive performance gaps arise not only from differences in family background but also—and more fundamentally—from disparities in school resources and educational quality (Castro and Rolleston, 2015). Long-term monitoring by India's National Sample Survey Office (NSSO) similarly reveals persistent educational inequality, with urban–rural disparities as a major driver and widening inequalities within rural areas over time (Agrawal, 2014).

China provides a particularly instructive case. Findings from large-scale datasets such as the China Family Panel Studies (CFPS) and the Rural–Urban Migration in China (RUMiC) survey consistently show that significant urban–rural educational gaps persist even after extensive statistical controls (Zhang et al., 2015). Drawing on data from the Chinese General Social Survey (CGSS), prior research documents cumulative urban–rural disparities across educational trajectories, indicating that rural hukou status and rural schooling are associated with lower probabilities of educational progression across schooling stages, particularly with respect to access to higher education (Hao et al., 2014). More recent studies find that urban students are significantly more likely to enter higher education, although this advantage does not uniformly translate into access to elite institutions (Song and Tan, 2022). Prior research also identifies several mechanisms underlying urban–rural gaps, including unequal allocation of school resources (Opoku-Asare and Siaw, 2015), family socioeconomic status (Zhao, 2011; Zhou, 2019), digital divides (Siaw et al., 2018; Wang et al., 2022), and teacher and peer effects (Burgis-Kasthala et al., 2018; Xu, 2023).

Across these bodies of literature, three major research gaps emerge. First, existing studies overwhelmingly focus on basic or secondary education (Bourdieu and Passeron, 1990; Qi and Zheng, 2019; Zheng et al., 2019), leaving the persistence of urban–rural disparities within higher education insufficiently examined. Although substantial disparities before university entry have been well documented (Agrawal, 2014; Hao et al., 2014; Castro and Rolleston, 2015; Zhang et al., 2015; Echazarra and Radinger, 2019; Cherry, 2021; Gorard et al., 2022), little is known about whether students from different backgrounds—once admitted to comparable universities with similar academic records—continue to diverge in their academic performance and developmental trajectories. Whether the hukou system exerts a latent influence within higher education should therefore be understood as a theoretically motivated yet empirically underexplored question, rather than as a mechanism that has been directly established at the tertiary level, particularly with respect to students' academic performance after university entry. Accordingly, the first research objective of this study is to conduct a systematic analysis within the context of higher education, aiming to contribute modestly to understanding whether urban–rural disparities persist after students enter university.

Second, while the literature frequently attributes urban–rural disparities to family background or school resources—such as resource accessibility (Farrington et al., 2015), teacher quality and class size (Ovenden-Hope and Passy, 2019), and family economic conditions (Zhao, 2011; Zhou, 2019)—much less attention has been paid to individual-level factors, particularly students' self-directed learning ability shaped by long-term exposure to unequal basic education environments. Constraints on rural students' early educational experiences may plausibly limit the development of effective learning strategies, beliefs, and competencies. This proposition is grounded in broader theories of skill formation and educational inequality and is presented as an interpretive framework rather than as direct empirical evidence specific to higher education. Accordingly, the second objective of this study is to incorporate students' self-directed learning ability into the mechanism analysis, building on existing research to provide a more comprehensive understanding of the multi-level pathways through which urban–rural disparities shape academic performance in higher education.

Third, from a methodological standpoint, much of the existing literature has relied on single-factor explanatory frameworks that examine the contribution of an isolated determinant to urban–rural disparities, while largely overlooking the potential interactions among multiple influencing factors. In contrast, this study adopts a multifactor analytical framework that simultaneously incorporates family economic status, parental educational attainment, and students' self-directed learning ability into a unified estimation model, treating these dimensions as mutually controlling covariates. Moreover, the empirical specification further introduces grade fixed effects and province fixed effects to systematically account for unobserved heterogeneity across academic stages and regional contexts, including differences in college entrance examination difficulty and admission policies. This approach strengthens the robustness and credibility of the empirical estimates and allows for a more nuanced identification of the multiple pathways through which urban–rural background jointly shapes academic performance in higher education, thereby contributing a more integrative and methodologically rigorous analytical perspective to the existing literature.

In sum, this study seeks to critically reassess whether and to what extent urban–rural background disparities persist in college students' academic performance within higher education, and to explore theoretically informed and associational pathways that may help interpret observed differences, within the constraints of an observational research design. Drawing on the theory of educational reproduction (Bourdieu, 1986), the study integrates family socioeconomic status, parental educational attainment, and students' self-directed learning ability within a unified analytical framework, thereby constructing a multifactorial model of influence. This approach aims to address existing gaps in the literature, particularly the limited empirical evidence and incomplete mechanistic explanations in the context of higher education, and to provide both theoretical and empirical insights into the generation and evolution of urban–rural educational disparities across educational stages.

## 2 Hypotheses

The influence of urban–rural background disparities on college students' academic outcomes constitutes a central issue in the study of educational equity and stratification. Bourdieu's theory of cultural reproduction posits that social class inequalities are perpetuated and magnified through the interaction of capital, habitus, and field, both across generations and within educational systems (Bourdieu, 1986). Specifically, economic capital provides the material foundation for educational investment, cultural capital shapes learning approaches and academic performance, and social capital expands educational opportunities through networks. Habitus, in turn, functions as an internalized disposition that drives individual learning behaviors. Within the university context—a distinctive educational field—students from urban and rural backgrounds differ substantially in family economic conditions, parental education, and self-regulated learning capacity, all of which directly or indirectly shape their academic outcomes (Zhang, 2023; Fu and Liu, 2024; Cheng, 2025).

### 2.1 Urban–rural disparities and academic outcomes

From the perspective of cultural reproduction, the educational field is not a neutral arena of fair competition but rather one deeply conditioned by unequal access to capital (Bourdieu, 1986). Urban families typically command greater economic and cultural capital, enabling them to provide richer learning resources, superior educational environments, and stronger academic support. In contrast, rural families often face significant financial and educational constraints. These disparities lead to distinct academic foundations and “habitus” even before students enter university, creating persistent differences in learning motivation, habits, and strategies (Zhao et al., 2023; Habib et al., 2024). Empirical evidence has consistently shown that urban students outperform their rural peers in academic achievement, self-efficacy, and learning engagement (Ma et al., 2023; Cheng, 2025). Building on this logic, we advance the following hypothesis:

*H1: Urban students demonstrate superior academic outcomes compared with their rural counterparts*.

### 2.2 The mediating role of parental education

Extending this argument, it is important to consider the role of parental education as an institutionalized form of cultural capital (Bourdieu, 1986). Parents with higher levels of education tend to hold greater expectations, adopt more effective educational strategies, and provide systematic guidance through knowledge transfer, methodological instruction, and value transmission, all of which positively influence students' academic outcomes (Zhou et al., 2021; Wang et al., 2022). Urban parents are more likely to create enriched home learning environments and foster autonomy and intrinsic motivation in their children, while rural parents, with comparatively lower levels of education, often rely on experiential or utilitarian approaches that provide weaker academic support (Wang et al., 2022). Thus, parental education may serve as a critical channel through which urban–rural disparities translate into differences in academic outcomes.

*H2: Parental education mediates the relationship between students' urban–rural background and their academic outcomes, such that urban students benefit from higher parental educational levels*.

### 2.3 The mediating role of family economic conditions

In addition to cultural capital, Bourdieu emphasizes that economic capital underpins other forms of capital and can be directly converted into educational resources and opportunities (Bourdieu, 1986). Urban–rural disparities in family economic conditions manifest most clearly in educational investment and learning environments. Urban families, with greater financial resources, are better able to provide tutoring, supplementary learning materials, and access to advanced educational technologies, thereby consolidating students' academic advantages (Ma et al., 2023; Fu and Liu, 2024). By contrast, rural families often face financial strain, which may compel students to work part-time or reduce investment in their education, ultimately constraining academic focus and outcomes (Zhang, 2023). Consequently, family economic conditions represent another pathway through which urban–rural disparities exert their influence.

*H3: Family economic conditions mediate the relationship between students' urban–rural background and their academic outcomes, such that urban students benefit from more favorable financial support*.

### 2.4 The mediating role of self-regulated learning ability

Beyond external resources, differences in habitus are also crucial. Bourdieu's concept of habitus underscores how long-term socialization produces stable cognitive patterns, behavioral tendencies, and value orientations that shape learning practices (Bourdieu, 1986). In the academic field, this is manifested in self-regulated learning ability, which encompasses goal setting, motivation, self-management, and the use of effective strategies (Gobel et al., 2013; Xu et al., 2021). Evidence indicates that urban students, benefiting from earlier exposure to diverse educational resources and structured learning approaches, tend to exhibit stronger self-regulated learning abilities. They are more adept at independently planning, acquiring knowledge proactively, and flexibly applying strategies. Conversely, rural students, often constrained by limited support, show weaker capacities in this regard and rely more heavily on external guidance (Fu and Liu, 2024; Cheng, 2025). Accordingly, self-regulated learning ability can be viewed as a key mediating mechanism linking urban–rural disparities to differences in academic outcomes.

*H4: Self-regulated learning ability mediates the relationship between students' urban–rural background and their academic outcomes, such that urban students' stronger learning autonomy contributes to superior outcomes*.

Taken together, and grounded in Bourdieu's theory of cultural reproduction, this study argues that urban–rural background disparities affect academic outcomes both directly—through differences in economic and cultural capital—and indirectly, via parental education (institutionalized cultural capital), family economic conditions (economic capital), and self-regulated learning ability (a manifestation of habitus). By integrating these perspectives, the framework not only clarifies the multidimensional mechanisms through which educational inequalities are reproduced, but also lays a robust theoretical foundation for the empirical analysis that follows.

## 3 Materials and methods

### 3.1 Data collection

Data for this study were collected in two phases, both implemented via online questionnaires. The research process adhered to academic research ethics, and all participants provided informed consent prior to participation. The survey was conducted in Anhui Province, China. Given that institutional tier and student quality may systematically influence academic performance, the study employed a stratified random sampling method with universities as the stratification units. Specifically, five first-tier universities in Anhui Province were selected as the survey sites. These universities have relatively similar college entrance examination admission thresholds, which helps ensure comparability in students' academic preparedness. Within each university, undergraduate students were randomly selected to participate in the survey.

The questionnaire was designed based on clear research objectives and core research questions, focusing on key measurement dimensions related to urban–rural background and students' academic outcomes. It comprised three main sections: (1) demographic characteristics, including household registration type, gender, and academic year; (2) family background, including family economic status and parental educational attainment; and (3) learning outcomes and individual learning ability. Learning outcomes were measured through academic ranking, number of certifications obtained, and on-campus awards, while items related to self-directed learning ability were also included. The questionnaire contained a total of 15 core measurement items. Following Hair et al. (2009), the recommended sample size for multivariate statistical analyses should be at least ten times the number of measurement items, yielding a minimum required sample of 150 for this study (Hair et al., 2009). Prior to formal administration, a small-scale pretest was conducted to identify ambiguous or poorly worded items, which were subsequently revised to enhance measurement validity and content adequacy.

The formal survey was distributed via the “Wenjuanxing” platform, which generated questionnaire links and QR codes for multi-channel dissemination to the target population. Respondents completed the questionnaires independently, with the survey homepage emphasizing anonymity and academic-use-only purposes to reduce social desirability bias and improve response authenticity. The first phase of data collection took place between April and June 2025, yielding 636 responses. To ensure data quality, invalid responses—such as those with unusually short completion times, missing key items, patterned or duplicate responses—were excluded, resulting in 498 valid questionnaires. The second phase was conducted in December 2025, producing 177 responses. Since 1st-year students had not yet completed final exams, their academic performance was not fully formed; thus, 1st-year responses were excluded. The remaining questionnaires were then subjected to the same quality control criteria as the first phase, removing responses with abnormal completion times, missing key information, or distorted response patterns, yielding 134 valid questionnaires. Combining both phases, the final dataset comprised 632 valid responses. Despite the difference in survey timing, the two phases targeted comparable student populations and followed identical survey instruments and sampling procedures. Regression results based on the pooled sample remain highly consistent with those obtained from the first-phase data, suggesting that the timing difference is unlikely to substantially affect the main conclusions. Overall, the sample size exceeds the minimum standards recommended in methodological literature, and the data quality is robust, providing a solid foundation for subsequent model estimation, mechanism analysis, and robustness checks.

Table 1 presents the demographic characteristics of the 632 valid responses. The distribution of household registration indicates that 330 students (52.2%) are from urban areas, and 302 students (47.8%) are from rural areas. Regarding gender, 287 respondents (45.4%) are female, and 345 (54.6%) are male. Academic year distribution includes 80 first-year students (12.6%), 136 2nd-year students (21.5%), 130 3rd-year students (20.6%), and 286 4th-year students (45.3%). Regarding only-child status, 239 students (37.8%) are only children, while 393 (62.2%) are not. Parental highest education levels are distributed as follows: junior high school or below 189 (29.9%), high school 129 (20.4%), junior college 105 (16.6%), undergraduate 139 (22.0%), and graduate or above 70 (11.1%). Overall, the sample demonstrates reasonable representation across urban–rural background, gender, academic year, and family background, providing a reliable basis for subsequent analyses.

**Table 1:** Demographic characteristics of the respondents.

**Characteristic**	**Category**	**Frequency**	**Percentage (%)**
Hukou	Urban	330	52.2
	Rural	302	47.8
Gender	Male	287	45.4
	Female	345	54.6
Grade	1st Year	80	12.6
	2nd Year	136	21.5
	3rd Year	130	20.6
	4th Year	286	45.3
Only Child	Yes	239	37.8
	No	393	62.2
Parental Education	Junior high or below	189	29.9
	Senior high	129	20.4
	Junior college	105	16.6
	Bachelor's degree	139	22.0
	Graduate or above	70	11.1

### 3.2 Variable definitions

#### 3.2.1 Dependent variable

College students' academic outcomes (L_ Outcomes) represent their tangible achievements in higher education in terms of knowledge acquisition, skill development, and overall competence (Biggs and Tang, 2007). In this study, academic outcomes are operationalized across three dimensions: academic ranking (L_ rank), number of professional certifications obtained (L_ certificate), and number of awards received during university (L_ award). Academic ranking directly reflects scholastic performance, certifications indicate professional skill accumulation, and awards capture broader personal development and active engagement. Integrating these dimensions allows for a comprehensive evaluation of students' learning outcomes. Within Bourdieu's theoretical framework, academic outcomes are viewed as the ultimate realization of how effectively a family's economic, cultural, and social capital is translated into educational success (Bourdieu, 1986).

#### 3.2.2 Independent variable

Household registration type (Urban) captures differences in social environment and educational opportunity (Bourdieu, 1986). Urban hukou students typically benefit from richer educational resources, stronger cultural environments, and enhanced access to high-quality schools and social networks. Conversely, rural hukou students may face disadvantages due to limited capital across economic, cultural, and social dimensions. Household registration is coded as a binary variable (urban = 1; rural = 0) to assess the direct effect of urban–rural background on learning outcomes, consistent with Bourdieu's notion of cultural reproduction, whereby social structural inequalities shape educational opportunities.

#### 3.2.3 Mediating variables

Family Economic Status (Economic): Reflecting Bourdieu's concept of economic capital (Bourdieu, 1986), family financial resources enable access to higher-quality educational inputs, stable learning environments, and enhanced psychological support. This variable is measured by annual household income: < RMB 30,000 = 1; RMB 30,000–50,000 = 2; RMB 50,000–100,000 = 3; > RMB 100,000 = 4.

Parental Education (Education): This variable operationalizes the family's cultural capital. Higher parental education is associated with more effective guidance on learning strategies, educational perspectives, and academic planning, thereby positively influencing students' outcomes (Bourdieu, 1986). Parental education is measured as the highest attained level: junior high school or below = 1, senior high school = 2, junior college = 3, bachelor's degree = 4, graduate degree or above = 5.

Self-Directed Learning Ability (Self-Directed): Referring to students' capacity to proactively set learning goals, design study plans, regulate their learning processes, and consistently execute learning tasks in the absence of external supervision, reflecting an integrated manifestation of learner agency and internalized cultural capital (Zimmerman, 2002). Given that this ability comprises multiple functional dimensions, it is operationalized in this study as a formative construct (Bollen and Lennox, 1991), encompassing three dimensions: learning strategies (LS), learning motivation (LM), and time management (TM) (Table 2). Students with higher self-directed learning ability are generally more adept at effectively allocating and utilizing learning resources, thereby attaining superior academic outcomes.

**Table 2:** Self-directed learning ability scale for college students.

**Dimension**	**Measurement item**	**Item no**.
Learning motivation (LM)	To what extent is your engagement in learning primarily driven by the goal of enhancing your competencies and achieving better personal development?	13
Learning strategies (LS)	To what extent are you able to proactively select and apply appropriate learning strategies in response to specific learning tasks?	14
Time management (TM)	To what extent are you able to effectively organize your daily study and extracurricular time and complete learning tasks as planned?	15

As a supplementary validation, exploratory factor analysis was conducted on these three indicators. Although self-directed learning is conceptualized as a formative construct, exploratory factor analysis is reported solely as a supplementary diagnostic tool to assess empirical coherence among indicators, rather than to imply the existence of an underlying latent variable. Results indicate that Bartlett's test of sphericity was significant (*p* < 0.001) and the KMO value was 0.68, reaching an acceptable level for empirical research. Moreover, all three indicators loaded onto a single common factor, demonstrating empirical consistency in capturing the same latent construct, thereby providing statistical support for the validity of the formative measurement. Crucially, in subsequent regression analyses and mediation tests, self-directed learning ability consistently exhibited a significant and robust positive association with students' learning outcomes (L_Outcomes). This association remained significant even after controlling for family background and other relevant covariates, aligning with prior research and indicating that the formative construct developed in this study possesses strong criterion-related validity in predicting external outcome variables.

#### 3.2.4 Control variables

To account for potential confounding factors, the study incorporates several control variables. Gender is coded as 1 for female students and 0 for male students, while Only Child Status is coded as 1 if the respondent is an only child and 0 otherwise, to control for the potential influence of gender differences and family structure on academic outcomes. Grade Level is measured using grade fixed effects (1st-year = 1, 2nd-year = 2, 3rd-year = 3, 4th-year = 4) to capture systematic differences across stages of study. In addition, province fixed effects are included to control for interprovincial heterogeneity in the National College Entrance Examination (Gaokao), including differences in examination difficulty and admission thresholds. All variable definitions and measurement details are summarized in Table 3.

**Table 3:** Variable definitions and measurements.

**Type**	**Variable**	**Symbol**	**Measurement**
Dependent variable	Academic outcomes	L_ Outcomes	Measured by a composite of academic ranking, number of professional certifications obtained, and on-campus awards received.
Independent variable	Household registration	Urban	Urban hukou coded as 1; rural hukou coded as 0.
Mediating variables	Family economic status	Economic	Measured by annual household income: < RMB 30,000 = 1; RMB 30,000–50,000 = 2; RMB 50,000–100,000 = 3; > RMB 100,000 = 4.
	Parental education	Education	Highest parental education level: junior high or below = 1; senior high school = 2; junior college = 3; bachelor's degree = 4; master's degree or above = 5.
	Self-regulated learning ability	Self_ Directed	Assessed based on learning strategies, learning motivation, and time management skills.
Control Variables	Gender	Gender	Female = 0; male = 1.
	Grade level	Grade	1st-year = 1; 2nd-year = 2; 3rd-year = 3; 4th-year = 4.
	Only child status	Only_ child	Yes = 1; No = 0.
	Province	Province	Dummy variable

### 3.2.5 Model specification

To examine the associational relationship between urban–rural background and college students' academic outcomes, we first specify the baseline regression model:


$$\begin{array}{l} L\text{\_}Outcomes=\beta_{0}+\beta_{1}Urban+\beta_{2}Gender+\beta_{3}Only_{child} \end{array}$$

$$\begin{array}{l} +\sum Grade_{j}+\sum Province_{k}+\varepsilon \end{array}$$ (1)

where *L*_*Outcomes* represents students' learning outcomes, and *Urban* is a dummy variable for urban household registration. The coefficient β_1_ captures the conditional association between urban–rural background and academic performance.

To explore the potential pathways through which urban–rural background is associated with learning outcomes, we consider three theoretically motivated mediating variables: family economic conditions (*Economic*), parental education (*Education*), and students' self-regulated learning ability (*Self*_*Directed*). The mediation pathways are tested through the following models:


$$\begin{array}{l} Mediator_{i}=\alpha_{0}+\alpha_{1}Urban+\beta_{2}Gender+\beta_{3}Only\text{\_}child \end{array}$$

$$\begin{array}{l} +\sum Grade_{j}+\sum Province_{k}+\gamma \end{array}$$ (2)

$$\begin{array}{l} L\_Outcomes=\beta_{0}^{'}+\beta_{1}^{'}Urban+\beta_{2}^{'}Mediator_{i}+\beta_{3}^{'}Gender\\ \;+\beta_{4}^{'}Only\_child+\sum Grade_{j}+\sum Province_{k}+\overset{'}{\varepsilon } \end{array}$$ (3)

In this framework, *Mediator*_*i*_ represents each mediator in turn. ∑*Grade*_*j*_ and ∑*Province*_*k*_ denote the inclusion of grade-level and province-level dummy variables in the model, respectively, to account for and mitigate the influence of inherent differences in learning outcomes among students from different academic years and provinces. Following the sequential testing approach proposed by Wen et al. (2004), a significant α_1_ in Model (2) combined with a significant $\beta_{2}^{/}$ in Model (3), is interpreted as evidence consistent with an indirect associational pathway linking urban–rural background to learning outcomes through the corresponding mediator (Wen et al., 2004).

## 4 Results

### 4.1 Correlation analysis

Table 4 reports the Pearson correlation matrix for the main variables. Overall, the correlation patterns are consistent with theoretical expectations and provide preliminary support for the subsequent regression analyses.

**Table 4:** Correlation analysis.

**Variables**		**1**	**2**	**3**	**4**	**5**	**6**	**7**	**8**
1	L_ Outcomes	1							
2	Urban	0.297^***^	1						
3	Education	0.282^***^	0.452^***^	1					
4	Economic	0.274^***^	0.315^***^	0.318^***^	1				
5	Self_ Directed	0.328^***^	0.330^***^	0.085^**^	0.154^***^	1			
6	Gender	0.021	0.114^***^	0.086^**^	0.047	−0.031	1		
7	Grade	0.088^**^	0.014	−0.022	0.321^***^	−0.031	−0.047	1	
8	Only_ child	0.033	0.191^***^	0.135^***^	0.022	0.148^***^	−0.016	−0.082^**^	1

^***^ and ^**^ denote statistical significance at the 1% and 5% levels, respectively; standard errors are in parentheses.

Learning outcomes (L_ Outcomes) are positively and significantly correlated with household registration status (Urban) (*r* = 0.297, *p* < 0.01), indicating that students from urban backgrounds exhibit higher academic performance than their rural counterparts. Learning outcomes are also positively associated with parental education (Education), family economic conditions (Economic), and self-directed learning ability (Self_ Directed), with correlation coefficients of 0.282, 0.274, and 0.328, respectively (all *p* < 0.01), suggesting that economic resources, cultural capital, and individual learning agency are closely related to academic performance.

Urban status is strongly correlated with parental education (*r* = 0.452, *p* < 0.01) and family economic conditions (*r* = 0.315, *p* < 0.01), indicating systematic urban advantages in family resources. It is also positively associated with self-directed learning ability (*r* = 0.330, *p* < 0.01), suggesting that differences in early-life environments are linked to variation in students' learning capacities.

Among control variables, gender shows no significant correlation with learning outcomes, while grade level is weakly but positively correlated (*r* = 0.088, *p* < 0.05). Only-child status is positively correlated with both urban status and parental education, consistent with China's institutional context. Multicollinearity is unlikely to be a concern, as all pairwise correlations are below 0.5 and variance inflation factors are below 3.

### 4.2 Urban–rural background disparities and academic outcomes

Table 5 reports the regression results examining the associational relationship between urban–rural background and university students' learning outcomes. In Model (1), with overall learning outcomes (L_ Outcomes) as the dependent variable and after controlling for gender and only-child status, as well as including grade fixed effects and province fixed effects, the coefficient on the urban household registration variable (Urban) is 1.215 and statistically significant at the 1% level. This result indicates that, holding other factors constant, students with an urban background exhibit significantly better overall learning outcomes than their rural counterparts. The finding indicates a statistically significant conditional association between urban–rural background and academic performance at the higher education stage. Accordingly, the results are consistent with Hypothesis 1, which posits persistent urban–rural disparities in learning outcomes among university students.

**Table 5:** Effect of urban–rural background disparities on college students' academic outcomes.

**Variables**	**(1)**	**(2)**	**(3)**	**(4)**
	**L_ Outcomes**	**L_ rank**	**L_ certificate**	**L_ award**
Urban	1.215^***^	0.337^***^	0.275^***^	0.604^***^
	(0.174)	(0.079)	(0.083)	(0.111)
Gender	−0.081	−0.025	−0.034	−0.023
	(0.170)	(0.077)	(0.081)	(0.108)
Only_ child	−0.263	−0.138^*^	−0.171^**^	0.045
	(0.182)	(0.083)	(0.087)	(0.116)
Constant	5.790^***^	3.354^***^	1.944^***^	0.492
	(0.708)	(0.322)	(0.337)	(0.451)
Grade FE	√	√	√	√
Province FE	√	√	√	√
N	632	632	632	632
R^2^	0.176	0.152	0.197	0.161

^***^, ^**^, and ^*^ denote statistical significance at the 1%, 5%, and 10% levels, respectively; standard errors are in parentheses.

Models (2) – (4) further examine different dimensions of learning outcomes by using academic rank (L_ rank), the number of professional certificates obtained (L_ certificate), and the number of on-campus awards received (L_ award) as dependent variables, respectively. The estimated coefficients for Urban are 0.337, 0.275, and 0.604, all of which are statistically significant at the 1% level. These results indicate that, relative to rural students, urban students not only outperform in academic achievement but also demonstrate clear advantages in professional skill accumulation (as reflected in certificate attainment) and overall performance quality (as reflected in awards received). Taken together, these findings suggest that the association between urban–rural background and learning outcomes is robust and manifests consistently across multiple dimensions of academic performance.

With respect to the control variables, the coefficients on gender (Gender) are not statistically significant across all model specifications, indicating that, conditional on other covariates, gender differences are not systematically associated with variations in learning outcomes. The only-child status variable (Only_ child) exhibits a negative and weakly significant or statistically significant association in the models for academic rank and certificate attainment, but remains insignificant in the models for overall learning outcomes and awards, pointing to heterogeneity across outcome dimensions. Importantly, the estimated coefficient on Urban remains statistically significant and consistent in sign across all specifications, underscoring the stability of the observed association.

In sum, the regression results provide clear evidence that, even after controlling for key structural differences such as grade level and province of origin, urban–rural background remains significantly associated with university students' learning outcomes. This empirical evidence supports the persistence of educational inequality at the higher education stage and lays a solid foundation for subsequent analyses of the potential explanatory pathways of family economic conditions, parental educational attainment, and students' self-directed learning ability in shaping learning outcomes.

### 4.3 Parental educational attainment as an associational pathway

This study employs the classical three-step regression approach to examine whether parental educational attainment is statistically associated with the relationship between urban–rural background and university students' learning outcomes. Specifically, Models (1) – (4) in Table 5 serve as the baseline regressions for Models (2) – (5) in Table 6, capturing the associations between urban–rural background and different dimensions of learning outcomes. Model (1) in Table 6 tests the effect of urban–rural background on the mediator, parental educational attainment. Models (2) – (5) in Table 6 then extend the corresponding baseline specifications by incorporating parental educational attainment to assess whether the observed urban–rural differences in learning outcomes are attenuated.

**Table 6:** Mediation effect of parental educational attainment.

**Variables**	**(1)**	**(2)**	**(3)**	**(4)**	**(5)**
	**Education**	**L_ Outcomes**	**L_ rank**	**L_ certificate**	**L_ award**
Urban	1.098^***^	0.841^***^	0.214^**^	0.288^***^	0.339^***^
	(0.096)	(0.189)	(0.087)	(0.092)	(0.120)
Education		0.341^***^	0.112^***^	−0.012	0.241^***^
		(0.073)	(0.033)	(0.035)	(0.046)
Gender	0.098	−0.114	−0.035	−0.033	−0.046
	(0.094)	(0.167)	(0.077)	(0.081)	(0.106)
Only_ child	0.044	−0.278	−0.143^*^	−0.170^**^	0.035
	(0.100)	(0.179)	(0.082)	(0.087)	(0.113)
Constant	2.229^***^	5.030^***^	3.104^***^	1.971^***^	−0.045
	(0.391)	(0.714)	(0.328)	(0.346)	(0.453)
Grade FE	√	√	√	√	√
Province FE	√	√	√	√	√
*N*	632	632	632	632	632
*R^2^*	0.271	0.205	0.168	0.197	0.198

^***^, ^**^, and ^*^ denote statistical significance at the 1%, 5%, and 10% levels, respectively; standard errors are in parentheses.

First, as shown in Model (1) of Table 6, the coefficient of Urban on parental educational attainment is positive and statistically significant (β = 1.098, *p* < 0.01), indicating that parents in urban households have significantly higher levels of education than those in rural households. This result satisfies a key statistical prerequisite commonly applied in mediation analyses, namely that the independent variable is systematically associated with the proposed mediator, and reflects structural urban–rural disparities in the accumulation of educational resources and cultural capital.

Second, the results reported in Table 5 show that urban–rural background is significantly associated with overall learning outcomes, academic rank, certificate attainment, and on-campus awards. These findings indicate that urban–rural disparities are associated with stable and robust direct effects across multiple dimensions of learning outcomes, thereby meeting the statistical criteria typically used to assess mediation patterns.

Third, in Models (2) – (5) of Table 6, which correspond to the baseline models in Table 5 and additionally include parental educational attainment, the mediator exhibits a significant positive association with students' learning outcomes. In particular, parental educational attainment is statistically significant in the models for overall learning outcomes, academic rank, and on-campus awards, suggesting that higher parental education is associated with superior academic performance and broader developmental outcomes in higher education. This pattern underscores the role of parental education as a key form of cultural capital.

More importantly, relative to the estimates in Table 5, the inclusion of parental educational attainment leads to a noticeable reduction in the magnitude of the Urban coefficient across all models. Although Urban remains statistically significant in some specifications, its effect size is clearly attenuated, indicating that part of the association between urban–rural background and learning outcomes is accounted for by parental educational attainment. This attenuation pattern suggests that parental educational attainment accounts for part of the observed association between urban–rural background and students' learning outcomes, which is consistent with a partial mediation interpretation in a statistical sense.

Taken together, the empirical results indicate that urban–rural background, parental educational attainment, and university students' learning outcomes are systematically related. Accordingly, Hypothesis 2 is supported. This evidence illuminates the underlying mechanism through which urban–rural educational disparities persist into higher education from the perspective of intergenerational transmission of cultural capital, and offers robust empirical support for the applicability of Bourdieu's theory of educational reproduction in the Chinese context.

### 4.4 Family economic conditions as an associational pathway

Beyond parental educational attainment, family economic conditions represent another important family-level dimension through which urban–rural background is statistically associated with university students' learning outcomes. To examine whether family economic conditions are systematically associated with the relationship between urban–rural background and learning outcomes, this study introduces family economic conditions as an intermediate explanatory variable and applies the classical three-step regression approach. The corresponding regression results are reported in Table 7.

**Table 7:** Mediation effect of family economic conditions.

**Variables**	**(1)**	**(2)**	**(3)**	**(4)**	**(5)**
	**Economic**	**L_ Outcomes**	**L_ rank**	**L_ certificate**	**L_ award**
Urban	0.611^***^	0.957^***^	0.256^***^	0.275^***^	0.426^***^
	(0.074)	(0.181)	(0.083)	(0.088)	(0.115)
Economic		0.423^***^	0.132^***^	0.000	0.291^***^
		(0.095)	(0.044)	(0.046)	(0.061)
Gender	0.048	−0.101	−0.031	−0.034	−0.036
	(0.072)	(0.167)	(0.077)	(0.081)	(0.106)
Only_child	−0.086	−0.227	−0.127	−0.171^**^	0.071
	(0.077)	(0.179)	(0.082)	(0.087)	(0.114)
Constant	2.134^***^	4.887^***^	3.072^***^	1.944^***^	−0.130
	(0.299)	(0.726)	(0.333)	(0.351)	(0.461)
Grade FE	√	√	√	√	√
Province FE	√	√	√	√	√
N	632	632	632	632	632
R^2^	0.298	0.202	0.165	0.197	0.192

^***^ and ^**^ denote statistical significance at the 1% and 5% levels, respectively; standard errors are in parentheses.

First, Model (1) in Table 7 examines the association between urban–rural background and family economic conditions. The results show that the coefficient of Urban is positive and statistically significant (β = 0.611, *p* < 0.01), indicating that students from urban households enjoy significantly more favorable economic resources than their rural counterparts. This finding indicates the presence of structural urban–rural disparities in economic capital and satisfies a commonly applied statistical prerequisite for examining mediation patterns.

Second, as evidenced by the baseline regressions reported in Table 5, urban–rural background is significantly associated with overall learning outcomes and their specific dimensions when the mediator is not included. This pattern indicates that urban–rural background is a stable predictor of learning outcomes, thereby meeting the statistical criteria typically used to assess mediation-related associations.

Third, in Models (2) – (5) of Table 7, family economic conditions are incorporated into the corresponding baseline specifications from Table 5. The results indicate that family economic conditions are positively and significantly associated with overall learning outcomes (L_Outcomes), academic rank (L_ rank), and on-campus awards (L_ award). These findings suggest that more favorable family economic conditions are associated with greater access to learning resources and support, which is, in turn, correlated with better academic performance. It is worth noting that although the coefficient of family economic conditions on certificate attainment (L_ certificate) is positive, it does not reach conventional levels of statistical significance. This implies that certificate acquisition may be more strongly shaped by factors such as field of study, individual career planning, and external institutional arrangements, rather than being determined solely by family economic resources.

At the same time, relative to the baseline results in Table 5, the coefficient of Urban declines noticeably across all specifications once family economic conditions are included. This attenuation pattern suggests that family economic conditions account for part of the observed association between urban–rural background and learning outcomes, in a statistical sense.

Taken together, the results indicate that family economic conditions constitute an important associational pathway linking urban–rural background to university students' learning outcomes, although the strength of this mechanism varies across different outcome dimensions. Urban–rural disparities are reflected in learning outcomes both directly and through their systematic association with the distribution of family economic resources, thereby exerting a sustained influence on students' learning investment and developmental opportunities. Accordingly, the empirical patterns observed in the data are consistent with Hypothesis H3. From the perspective of economic capital, this finding complements and extends the analysis of cultural capital pathways presented earlier, jointly revealing the multidimensional transmission structure through which family background continues to shape educational outcomes in higher education.

### 4.5 Self-directed learning ability as an associational pathway

Beyond family background factors, individual-level differences in learning ability may also represent an important dimension through which urban–rural background is statistically associated with university students' learning outcomes. Accordingly, this study further incorporates self-directed learning ability into the analytical framework and examines its role as an associational pathway linking urban–rural disparities to learning outcomes. The relevant results are reported in Table 8, where Models (1) – (4) in Table 5 serve as the baseline specifications for Models (2) – (5) in Table 8.

**Table 8:** Mediation effect of self-directed learning ability.

**Variables**	**(1)**	**(2)**	**(3)**	**(4)**	**(5)**
	**Self_ Directed**	**L_ Outcomes**	**L_ rank**	**L_ certificate**	**L_ award**
Urban	1.326^***^	0.864^***^	0.209^**^	0.166^*^	0.489^***^
	(0.158)	(0.179)	(0.082)	(0.087)	(0.116)
Self_ Directed		0.265^***^	0.096^***^	0.082^***^	0.086^***^
		(0.044)	(0.020)	(0.021)	(0.028)
Gender	−0.299^*^	−0.002	0.004	−0.009	0.003
	(0.154)	(0.166)	(0.076)	(0.080)	(0.108)
Only_child	0.312^*^	−0.346^*^	−0.168^**^	−0.196^**^	0.019
	(0.165)	(0.177)	(0.081)	(0.086)	(0.115)
Constant	9.627^***^	3.241^***^	2.427^***^	1.154^***^	−0.340
	(0.642)	(0.807)	(0.371)	(0.391)	(0.525)
Grade FE	√	√	√	√	√
Province FE	√	√	√	√	√
N	632	632	632	632	632
R2	0.226	0.223	0.184	0.216	0.174

^***^, ^**^, and ^*^ denote statistical significance at the 1%, 5%, and 10% levels, respectively; standard errors are in parentheses.

First, Model (1) uses self-directed learning ability (Self_ Directed) as the dependent variable. The regression results show that the coefficient of the urban–rural background variable (Urban) is positive and statistically significant (β = 1.326, *p* < 0.01), indicating that, compared with their rural counterparts, urban students are statistically associated with higher levels of self-directed learning in terms of learning motivation, learning strategies, and time management. This finding suggests that urban–rural disparities are not confined to differences in family resources but are also reflected in students' individual learning behaviors and self-regulatory capacities during the university stage.

Second, when self-directed learning ability is introduced into the learning outcome regressions in Models (2) – (5), it is positively and significantly associated with overall learning outcomes (L_ Outcomes) as well as with specific dimensions, including academic rank, the number of certificates obtained, and the frequency of on-campus awards. The estimates are highly consistent across different outcome measures, indicating that self-directed learning ability represents an important individual-level correlate of disparities in university students' learning outcomes.

Further comparison of the results in Tables 5, 8 reveals that, after controlling for self-directed learning ability, the regression coefficients of the urban–rural background variable decline markedly across all specifications relative to the baseline regressions. This pattern suggests that self-directed learning ability accounts for part of the observed association between urban–rural background and learning outcomes in a statistical sense. Taken together with the significant association between urban–rural background and self-directed learning ability, as well as the robust association between self-directed learning ability and learning outcomes, these results indicate that self-directed learning ability constitutes an important associational pathway linking urban–rural background to university students' learning outcomes.

In contrast to the family-level dimensions reflected by family economic conditions and parental educational attainment, self-directed learning ability captures an individual-level dimension through which urban–rural disparities are reflected in students' learning behaviors and competence structures at the higher education stage. These results suggest that urban–rural background is associated with learning outcomes not only through family capital but also through systematic differences in students' approaches to learning and self-regulatory capacities. Accordingly, the empirical patterns observed in the data are consistent with Hypothesis H4 and extend existing discussions of urban–rural educational disparities from the perspective of individual ability formation.

### 4.6 Multiple mediating pathways

To provide a comprehensive account of the mechanisms through which urban–rural background influences university students' learning outcomes, this study simultaneously incorporates three mediating variables—parental educational attainment (Education), family economic conditions (Economic), and self-directed learning ability (Self_ Directed)—within a unified regression framework. The corresponding results are reported in Table 9. Models (1) – (4) in Table 5 serve as the baseline specifications for each outcome variable, while Models (1) in Tables 6–8 are used to examine the associations between urban–rural background and the respective mediators.

**Table 9:** Multiple mediation effects.

**Variables**	**(1)**	**(2)**	**(3)**	**(4)**
	**L_ Outcomes**	**L_ rank**	**L_ certificate**	**L_ award**
Urban	0.332^*^	0.035	0.175^*^	0.122
	(0.194)	(0.090)	(0.097)	(0.126)
Education	0.321^***^	0.112^***^	−0.005	0.214^***^
	(0.072)	(0.034)	(0.036)	(0.047)
Economic	0.294^***^	0.085^*^	−0.007	0.216^***^
	(0.094)	(0.044)	(0.047)	(0.061)
Self_Directed	0.263^***^	0.095^***^	0.082^***^	0.086^***^
	(0.043)	(0.020)	(0.021)	(0.028)
Gender	−0.044	−0.009	−0.009	−0.026
	(0.161)	(0.075)	(0.080)	(0.104)
Only_child	−0.320^*^	−0.156^*^	−0.197^**^	0.033
	(0.172)	(0.080)	(0.086)	(0.112)
Constant	1.912^**^	2.011^***^	1.177^***^	−1.276^**^
	(0.813)	(0.379)	(0.407)	(0.528)
Grade FE	√	√	√	√
Province FE	√	√	√	√
N	632	632	632	632
R2	0.274	0.217	0.216	0.231

^***^, ^**^, and ^*^ denote statistical significance at the 1%, 5%, and 10% levels, respectively; standard errors are in parentheses.

As shown in Table 9, all three mediators are positively and significantly associated with learning outcomes, albeit with notable heterogeneity across dimensions. Parental educational attainment has a significantly positive impact on overall learning outcomes (L_ Outcomes), academic rank (L_ rank), and on-campus awards (L_ award), indicating that cultural capital remains an important correlational pathway linked to academic achievement. Family economic conditions also significantly affect L_ Outcomes, L_ rank, and L_ award, reflecting the supportive role of economic resources in overall academic performance. However, neither parental education nor family economic conditions exhibit a statistically significant association with certificate acquisition (L_ certificate), suggesting that the attainment of professional skill certificates may be less directly connected to family background characteristics. In contrast, self-directed learning ability shows a consistently positive and statistically significant association across all outcome dimensions, underscoring the close linkage between proactive learning behaviors and academic performance.

After controlling for all three mediators, the coefficient associated with urban–rural background is substantially attenuated (for example, the coefficient for L_ Outcomes declines from 1.215 in the baseline regression in Table 5 to 0.332 in Table 9) and becomes statistically insignificant for some outcome dimensions. This attenuation pattern suggests that the observed association between urban–rural background and learning outcomes is partly accounted for by parental educational attainment, family economic conditions, and self-directed learning ability. Rather than implying a strict causal decomposition, these results indicate that urban–rural background, family resources, and individual learning abilities are interrelated dimensions that jointly characterize disparities in students' academic performance. The relative contribution of each pathway varies across outcome dimensions, but together they form a complementary set of associational channels linking background characteristics to learning outcomes.

In sum, the multiple mediation model not only corroborates the patterns identified in the preceding single-mediator analyses but also highlights the interconnected roles of family background and individual learning characteristics in shaping academic outcomes. Given the cross-sectional nature of the data, these findings should be interpreted as evidence of structured associations rather than definitive causal pathways. In particular, reciprocal or endogenous relationships—especially between self-directed learning ability and academic outcomes—cannot be fully ruled out. Nevertheless, the results provide a more nuanced empirical account of how urban–rural disparities are linked to educational outcomes through a combination of resource endowments and individual learning orientations, thereby enriching the understanding of multidimensional inequality processes within higher education.

### 4.7 Robustness checks

#### 4.7.1 Bootstrap mediation analysis

To assess the robustness of the mediation results, this study re-examines the mediating effects using a nonparametric bootstrap approach. Specifically, 5,000 resamples are drawn, and 95% confidence intervals are constructed based on the bias-corrected percentile method. Consistent with the cross-sectional nature of the data, this procedure is employed to evaluate the statistical stability of the estimated indirect associations rather than to establish causal ordering among variables.

The results indicate that the indirect effects along all three associational mediating pathways—parental educational attainment, family economic conditions, and self-directed learning ability—are statistically significant, with none of the corresponding 95% confidence intervals containing zero. Moreover, both the total indirect effect and the direct effect remain statistically significant, with signs and magnitudes consistent with those obtained from the baseline regressions and the stepwise mediation tests. These results suggest that urban–rural background is systematically associated with university students' learning outcomes through multiple interrelated channels, while not precluding the possibility of reciprocal or endogenous relationships among the mediators and learning outcomes.

Accordingly, the bootstrap analysis provides additional evidence for the robustness of the observed associational pathways, rather than definitive proof of causal mediation. The bootstrap results are reported in Table 10.

**Table 10:** Bootstrap mediation analysis.

**Paths**	**Observed coefficient**	**Bootstrap std. err**.	***z***	***P*>|*z*|**	**95%CI (lower)**	**95%CI (upper)**
ind_edu	0.345	0.089	3.870	0.000	0.170	0.519
ind_eco	0.211	0.062	3.380	0.001	0.089	0.333
ind_self	0.379	0.072	5.250	0.000	0.238	0.521
total_ind	0.935	0.116	8.040	0.000	0.707	1.162
direct	0.407	0.193	2.110	0.035	0.029	0.785
total	1.341	0.168	7.980	0.000	1.012	1.671

Variables in the *Paths* column are defined as follows: ind_edu denotes the indirect effect mediated by parental educational attainment; ind_eco denotes the indirect effect mediated by family economic conditions; ind_self denotes the indirect effect mediated by self-directed learning ability; total_ind represents the total indirect effect of the three mediators combined; direct refers to the direct effect after controlling for the mediating variables; and total indicates the total effect of urban–rural background on learning outcomes.

#### 4.7.2 Sensitivity analysis

In addition, to examine potential biases arising from treating ordered variables as continuous, a sensitivity analysis is conducted by re-estimating the models using dummy-variable coding for parental educational attainment and family economic conditions. The corresponding regression results are presented in Table 11. Column (1) reports the baseline model, which includes only urban–rural background and control variables. Column (2) augments this specification by adding categorical dummy variables for parental educational attainment. Column (3) further introduces dummy variables for family economic conditions. Column (4) simultaneously incorporates all three mediators, including parental education, family economic conditions, and self-directed learning ability.

**Table 11:** Sensitivity analysis.

**Variables**	**(1)**	**(2)**	**(3)**	**(4)**
	**L_ Outcomes**	**L_ Outcomes**	**L_ Outcomes**	**L_ Outcomes**
Urban	1.215^***^	0.809^***^	0.949^***^	0.318
	(0.174)	(0.189)	(0.180)	(0.195)
Edu2		0.745^***^		0.709^***^
		(0.230)		(0.222)
Edu3		0.571^**^		0.577^**^
		(0.264)		(0.254)
Edu4		1.344^***^		1.213^***^
		(0.254)		(0.253)
Edu5		1.221^***^		1.183^***^
		(0.392)		(0.387)
Eco2			0.414^*^	0.399^*^
			(0.242)	(0.233)
Eco3			0.439^*^	0.283
			(0.250)	(0.243)
Eco4			1.474^***^	1.050^***^
			(0.300)	(0.297)
Self_ Directed				0.252^***^
				(0.043)
Gender	−0.081	−0.119	−0.114	−0.063
	(0.170)	(0.167)	(0.167)	(0.161)
Only_ child	−0.263	−0.240	−0.188	−0.274
	(0.182)	(0.178)	(0.179)	(0.172)
Constant	5.790^***^	6.416^***^	6.357^***^	3.646^***^
	(0.708)	(0.715)	(0.708)	(0.808)
Grade FE	√	√	√	√
Province FE	√	√	√	√
N	632	632	632	632
R^2^	0.176	0.221	0.217	0.287

Edu2–Edu5 are dummy variables for parental educational attainment (Education), with Edu1 serving as the reference category; Eco2–Eco4 are dummy variables for family economic conditions (Economic), with Eco1 as the reference category. ^***^, ^**^, and ^*^ denote statistical significance at the 1%, 5%, and 10% levels, respectively; standard errors are in parentheses.

The results show that, in the baseline model, urban–rural background (Urban) has a significantly positive association with learning outcomes. After introducing the parental education dummies (Column 2), the coefficient on Urban declines substantially but remains statistically significant, while the coefficients on the parental education categories are jointly positive and significant, increasing monotonically with higher educational levels. This pattern suggests that parental educational attainment is an important associational pathway linking urban–rural background and learning outcomes. When family economic condition dummies are further included (Column 3), the coefficient on Urban decreases again, and the highest economic category (Eco4) exhibits a significantly positive association with learning outcomes, suggesting that family economic resources constitute another relevant associational transmission channel.

When parental educational attainment, family economic conditions, and self-directed learning ability are simultaneously controlled for (Column 4), the coefficient on Urban becomes statistically insignificant, whereas self-directed learning ability continues to exert a significant and positive association on learning outcomes. Consistent with the cross-sectional design, this result should be interpreted as indicating that observed urban–rural disparities in learning outcomes are largely correlated with differences in family background and individual capability characteristics, rather than as evidence of a fully specified causal mechanism. Overall, after relaxing the linearity assumption for ordered variables and adopting dummy-variable coding, the directions and significance levels of the mediators, as well as the pattern of changes in the Urban coefficient, remain consistent with the baseline results, providing additional evidence for the robustness of the observed associational relationships rather than definitive causal claims.

## 5 Discussion

### 5.1 Findings

Based on an empirical analysis of a sample of enrolled university students with comparable Gaokao examination scores, this study re-examines the association between urban–rural background and learning outcomes at the higher education stage and further investigates the mediating roles of family economic conditions, parental educational attainment, and students' self-directed learning ability. The main conclusions are as follows.

First, after controlling for Gaokao performance, grade fixed effects, and province fixed effects, urban–rural background differences in university learning outcomes remain statistically significant. Overall, students from urban backgrounds exhibit significantly higher levels of learning outcomes than their rural counterparts. This finding suggests that even when urban and rural students enter the same or similarly ranked universities with comparable academic credentials, background-related disparities may continue to operate in more implicit ways during the university stage. This result is consistent with prior research documenting the persistence of educational inequality across educational stages (Hao et al., 2014; Zhang et al., 2015; Reay, 2018) and provides complementary evidence from the context of higher education.

Second, the mechanism analysis indicates that family economic conditions and parental educational attainment play important mediating roles in the relationship between urban–rural background and university learning outcomes. Compared with students from rural backgrounds, those from urban backgrounds are generally more advantaged in terms of household economic resources and parental educational capital. These advantages are stably associated with greater access to learning resources, more effective forms of academic support, and higher educational expectations. This finding accords with cultural reproduction theory, which emphasizes the enduring influence of family background on educational outcomes (Bourdieu, 1986), and is also consistent with both international and Chinese studies documenting the impact of family socioeconomic status on performance in higher education (Lareau, 2011; Zhao, 2011).

Third, self-directed learning ability is found to be significantly and robustly positively associated with university learning outcomes. The inclusion of self-directed learning ability in multivariate models substantially improves explanatory power. Although the direct association between urban–rural background and self-directed learning ability is relatively limited, the results indicate that, within existing family and institutional constraints, individual-level learning competencies and strategies remain closely linked to academic performance. This finding is in line with research on self-regulated learning theory (Zimmerman, 2002) and resonates with studies suggesting that learner agency may play a constructive role in partially mitigating structural disadvantages.

Taken together, the results demonstrate that the influence of urban–rural background on learning outcomes in higher education is not driven by a single factor, but rather operates through multiple pathways, including family economic conditions, parental educational attainment, and individual learning ability. By integrating family-, individual-, and institutional-level factors into a unified analytical framework, this study provides more systematic empirical evidence for understanding how urban–rural educational disparities manifest and persist at the university stage.

### 5.2 Implications

At the theoretical level, this study contributes to the literature on the sociology of education and educational inequality in several respects. First, it extends the analytical focus on urban–rural disparities to the higher education stage, thereby addressing the relative lack of attention in prior research to whether such disparities persist after students enter university. The findings demonstrate that urban–rural differences are not fully attenuated by access to higher education; rather, they continue to shape learning outcomes through the intertwined influences of family background and individual capabilities. In this regard, the study provides additional empirical support for reproduction theory in the context of higher education (Bourdieu and Passeron, 1990; Reay, 2018).

Second, by simultaneously incorporating family economic conditions, parental educational attainment, and self-directed learning ability into the mechanism analysis, this study moves beyond the single-factor explanatory frameworks commonly adopted in earlier research. Compared with studies that focus exclusively on economic resources or parental education (Davis-Kean, 2005; Wu, 2010), the multi-path analytical approach adopted here more precisely elucidates how structural conditions and individual characteristics jointly contribute to urban–rural disparities. This integrated perspective helps deepen understanding of the complex and multifaceted processes through which educational inequality is produced and reproduced.

At the practical level, the findings offer several implications for advancing equity in higher education. First, the continued association between urban–rural background and learning outcomes at the university stage suggests that institutions should remain attentive to the relative disadvantages faced by students from rural backgrounds in terms of family resources when allocating educational resources and designing student support systems. Second, the robust link between self-directed learning ability and academic outcomes indicates that targeted interventions—such as curriculum design, pedagogical reforms, and learning support programs aimed at strengthening students' learning strategies and competencies—may help mitigate the adverse effects associated with family background differences. Overall, promoting equity in higher education requires coordinated efforts at both the structural level of resource provision and the individual level of capacity development.

### 5.3 Limitations

Several limitations should be acknowledged. First, data availability imposes certain constraints. The sample is drawn from five top-tier universities within a single Chinese province, which are relatively comparable in terms of admission thresholds, size, and overall ranking. While this sampling frame suffices for the current analysis, it overlooks variations across institutions of different tiers and geographic regions, potentially limiting the generalizability of the findings. Moreover, the concentration of the sample within specific universities precludes the identification or control of potential institution-level clustering effects. Additionally, the study relies on cross-sectional data, which constrains the ability to establish causal relationships among the variables. Future research could expand the sample to include universities across multiple regions and tiers, employ multilevel modeling or cluster-robust estimation techniques, and integrate longitudinal data or quasi-experimental designs to enhance the robustness, generalizability, and causal interpretability of the results.

Second, the explanation of underlying mechanisms requires further refinement. This study examines mediation through parental educational attainment, family economic conditions, and self-directed learning ability, providing an initial understanding of the transmission pathways of urban–rural disparities. However, other potential factors, such as students' psychological capital or social support networks, may also play critical roles. Future research should incorporate additional sociological and psychological variables to construct a more comprehensive model of the mechanisms driving these disparities.

Finally, this study primarily emphasizes empirical testing of education reproduction theory, highlighting the persistence of urban–rural disparities in higher education. How educational policies and institutional interventions can effectively mitigate these disparities remains an open question. Future work could examine specific policy initiatives and university-level interventions to provide more in-depth insights into pathways for achieving equity in higher education.

## Data Availability

The raw data supporting the conclusions of this article will be made available by the authors, without undue reservation.
